# Conformational effects of a common codon 751 polymorphism on the C-terminal domain of the xeroderma pigmentosum D protein

**DOI:** 10.4103/1477-3163.54918

**Published:** 2009-08-06

**Authors:** Regina Monaco, Ramon Rosal, Michael A. Dolan, Matthew R. Pincus, Greg Freyer, Paul W. Brandt-Rauf

**Affiliations:** 1 Department of Environmental Health Sciences, Mailman School of Public Health, Columbia University, New York, NY 10032 USA; 2 Chemical Terrorism Laboratory, New York City Department of Health and Mental Hygiene, New York, NY 10016 USA; 3 Tripos, Inc., St. Louis, MO 63144 USA; 4 Department of Pathology and Laboratory Medicine, New York Harbor VA Medical Center, Brooklyn, NY 12209 USA; 5 Division of Environmental and Occupational Health Sciences, School of Public Health, University of Illinois at Chicago, Chicago, IL 60612 USA

**Keywords:** Genetic polymorphism, structure-function correlation, nucleotide excision repair, molecular dynamics

## Abstract

**Aim:**

The xeroderma pigmentosum D (XPD) protein is a DNA helicase involved in the repair of DNA damage, including nucleotide excision repair (NER) and transcription-coupled repair (TCR). The C-terminal domain of XPD has been implicated in interactions with other components of the TFIIH complex, and it is also the site of a common genetic polymorphism in XPD at amino acid residue 751 (Lys->Gln). Some evidence suggests that this polymorphism may alter DNA repair capacity and increase cancer risk. The aim of this study was to investigate whether these effects could be attributable to conformational changes in XPD induced by the polymorphism.

**Materials and Methods:**

Molecular dynamics techniques were used to predict the structure of the wild-type and polymorphic forms of the C-terminal domain of XPD and differences in structure produced by the polymorphic substitution were determined.

**Results:**

The results indicate that, although the general configuration of both proteins is similar, the substitution produces a significant conformational change immediately N-terminal to the site of the polymorphism.

**Conclusion:**

These results provide support for the hypothesis that this polymorphism in XPD could affect DNA repair capability, and hence cancer risk, by altering the structure of the C-terminal domain.

## INTRODUCTION

The XPD (xeroderma pigmentosum D) protein (also called ERCC2 or excision repair cross-complementing group 2 protein) is a key component of the cellular machinery responsible for nucleotide excision repair (NER) and transcription-coupled repair (TCR) of DNA damage.[1] Xeroderma pigmentosum D is a DNA helicase that functions as a subunit of the transcription factor IIH complex (TFIIH) to promote DNA bubble formation at the damaged site by unwinding the DNA in preparation for the subsequent steps in the repair process.[1] In order to accomplish this, XPD is assumed to require the appropriate conformation to interact with the DNA substrate and other components of the TFIIH complex.

The C-terminal domain of XPD is the site of interaction with the p44 helicase activator protein of the TFIIH complex.[2] The C-terminal domain is also the site of a very common single nucleotide polymorphism that results in the substitution of a glutamine (Gln) for the normally occurring lysine (Lys) at amino acid residue 751.[3] Although the data to date have been inconsistent, some evidence from both epidemiologic and experimental studies suggests that this polymorphism in XPD may alter DNA repair capabilities and cancer risk.[4] Thus, it is theoretically possible that this polymorphism is responsible for altering the structure of the C-terminal domain and hence the function of the XPD protein by disrupting critical protein-protein interactions and affecting its role in DNA repair. The purpose of the present study was to investigate this possibility by determining if there are differences in the structure of the C-terminal domain with Lys and Gln at position 751 using molecular dynamics techniques.

## MATERIALS AND METHODS

The starting structure for determining the conformational effect of the substitution of Gln for Lys at amino acid residue 751 of XPD is the previously constructed model of the wild-type XPD protein.[5] This model for wild-type XPD was developed based primarily on the structural and functional relationship of the protein with a bacterial NER protein UvrB, for which there is a known X-ray crystallographic structure. However, since the C-terminal XPD sequence (amino acid residues 713–761) did not exhibit high sequence homology with the UvrB C-terminus, this portion of the model was developed by a search for a structurally similar folded homolog. The search identified 4HB1, a designed four-helical bundle, as the best fit, where only the coordinates from two of the helices were used as the model for the XPD C-terminus. In this resultant model, the fold of the XPD C-terminus was shown to be similar to that of UvrB.[5] The coordinates for this wild-type XPD structure were obtained (Courtesy of Drs. Bennett Van Houten and Rachelle J. Bienstock of NIEHS) and the coordinates for the C-terminus from amino acid residues Leu 721 to Leu 760 were used as the starting point for our computations. This structure was missing two amino acid side chain groups (Lys 751 and Gln 759), which were first added and then optimized using standard minimization techniques to relax the position of these side chain groups and remove steric clashes. Our further refinement of this structure for the wild-type C-terminal domain with Lys at amino acid residue 751 and computation of the structure of the polymorphic C-terminal domain with Gln at amino acid residue 751 relied on an adaptation of a molecular dynamics approach as previously described for other protein structural determinations, which has been shown to yield results that are consistent with experimental data.[6–8]

First, each amino acid residue in the XPD structure was relaxed and minimized using Sybyl's Structure Preparation Tool (Sybyl; Tripos, St. Louis, MO), which allowed steric clashes to be removed. Then the charges of the C-terminal domain were neutralized by the random placement of 4 Cl^−^ and 7 Na^+^ counter ions. These counter ions were allowed to relax individually to correspond to physico-chemically reasonable placement. The molecule was then immersed in a water box of 17 183 water molecules and the water molecules were also allowed to relax while the XPD structure was held restrained.

A series of nested energy minimizations were then performed on this complex of the XPD molecule, counter ions and water, resulting in an overall minimized structure for the wild-type C-terminal domain. After the overall structure was minimized to a root mean square (RMS) gradient of ≤0.05 A, the dynamics runs were carried out. The XPD complex was heated to 300°K over 2 ps and then allowed to equilibrate over the balance of the dynamics run. The energies, volume and density of the completed dynamics run were examined to insure that the results were physically reasonable. A final average wild-type XPD structure was calculated from the energy-equilibrated structures (frames) generated during the final 25–50 ps of the run.

This same procedure was followed to determine the structure of the polymorphic C-terminal domain with Gln substituted for the normally occurring Lys at amino acid residue 751 yielding a final average polymorphic XPD structure. Finally, the average structure for the polymorphic protein was superimposed on that for the wild-type protein such that the RMS deviation of the coordinates of the backbone atoms of one structure from the other was a minimum. The average RMS deviation between the polymorphic and wild-type proteins was determined for the structures as a whole, as well as for each amino acid residue individually to identify isolated regions with the most significant conformational changes.

## RESULTS

**Figure 1:**
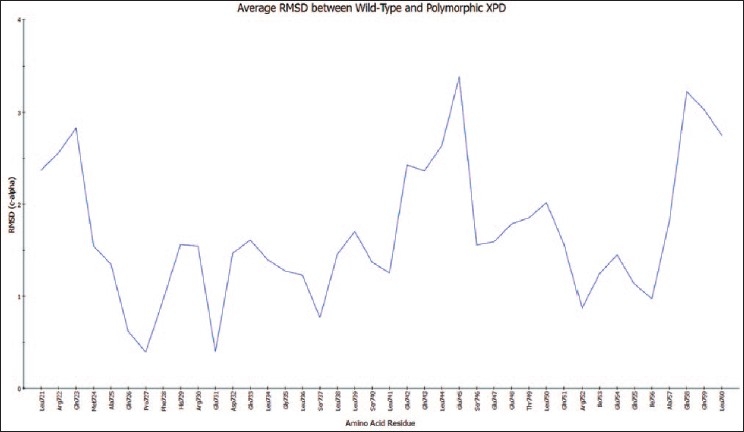
Individual residue backbone deviations for the coordinates of corresponding amino acid residues 721–760 of the average structure of the polymorphic XPD C-terminal domain from those of the wild-type domain average structure. Scale is in angstroms

The overall RMS deviation for the average structures of the wild-type and polymorphic proteins was only 1.84 A, and the general configuration of the wild-type C-terminal domain was retained in the polymorphic form. However, the individual residue RMS deviations for the average structures are shown in Figure 1, demonstrating a moderately large deviation (>3 A) around amino acid residues Gln 743 to Ser 746. Interestingly, at the site of the polymorphism at residue 751, there is a minimal deviation (1.5 A) between the two structures.

**Figure 2:**
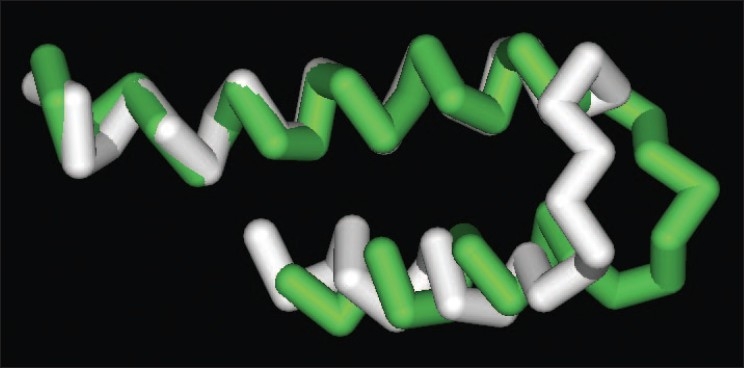
Superposition of the Cα tracings of the average structures of the wild-type (white) and polymorphic (green) forms of the C-terminal domain of XPD. The N-termini are to the upper left

Figure 2 shows the best-fit superposition of the average backbone structures for the C-terminal domains of the wild-type (white) and the polymorphic (green) proteins. As this figure demonstrates, the general configuration of the wild-type protein was retained in the polymorphic form. Both structures can be seen to have two α-helices separated by a bend at amino acid residues Ser 740 to Leu 750, and it is in the bend region immediately N-terminal to the site of the polymorphism that the two structures differ, particularly at residues Gln 743 to Ser 746. The bend in the wild-type structure is essentially outside the plane of the two flanking helical regions, approximately perpendicular to them, whereas in the polymorphic form the bend lies more directly within the plane of the two flanking helices.

## DISCUSSION

These results suggest that the polymorphic substitution of Gln for Lys at amino acid residue 751 in the XPD protein could produce a discrete and potentially significant conformational change in the C-terminal domain. Although the site of the substitution itself does not show much deviation in the two structures, the substitution appears to be responsible for a deviation in the bend region immediately N-terminal to the site of the polymorphism.

The conformational effects noted in this study could be consistent with other lines of evidence. First, the importance of the C-terminus to XPD function is underscored by the fact that a deletion mutation resulting in the loss of the final 17 C-terminal amino acids, including residue 751, is known to cause the clinical phenotype of trichothiodystrophy, which is characterized by deficiencies in NER[9]; curiously, trichothiodystrophy is not generally associated with an increased risk of cancer, although this may be at least partially attributable to the fact that patients tend to die young of other causes.

As noted above, the specific effects of the XPD Gln751 polymorphism have been investigated in numerous epidemiologic and a few experimental studies; however, the results as a whole have not been consistent. For example, several experimental studies involving the *in vitro* exposure of cultured lymphocytes to mutagenic agents such as UV light or X-rays have found statistically significant decreased DNA repair capability in cells with the XPD Gln751 polymorphism.[10–12] On the other hand, an *in vitro* study using reconstituted recombinant TFIIH complexes containing different XPD variants found no significant differences in helicase activity, ATPase activity or basal transcription ability in complexes with the XPD Gln751 polymorphism.[13] It is possible that these discrepancies are due at least in part to differences in the type of DNA damage examined or the sensitivities of the assays employed. Similarly, in terms of epidemiologic studies, a recent meta-analysis of 56 case-control studies found only a small, albeit statistically significant, overall increased risk for all cancers for the 751 Gln/Gln genotype compared to the Lys/Lys genotype (OR = 1.10, 95%CI = 1.03–1.16).[14] However, for certain specific cancers the risks for the Gln/Gln genotype compared to the Lys/Lys genotype were considerably greater and with more robust statistical significance, particularly esophageal cancer (OR = 1.61, 95%CI = 1.16–2.25) and acute lymphoblastic leukemia (OR=1.83, 95%CI=1.21–2.75).[14] It is possible that some of these discrepancies in the epidemiologic literature are due to variable effects of the polymorphism depending on the cancer site investigated and the different risk factors contributing to the different cancers. At any rate, it can be said that at least in some studies, the XPD Gln751 polymorphism has been associated with diminished DNA repair capability and an increased risk of cancer.

Data from our own prior epidemiologic studies, which prompted the current research, also suggest that the XPD Gln751 polymorphism leads to a reduction in DNA repair capability *in vivo.*[15] For example, we have studied a model population of workers exposed to the known mutagen/carcinogen vinyl chloride and the effect of this XPD polymorphism on the occurrence of biomarkers of mutations in this cohort. Vinyl chloride is known to be metabolized to the reactive intermediates chloroethylene oxide and chloroacetaldehyde, which form promutagenic etheno-DNA adducts. The resultant etheno-guanine DNA adduct is believed to be responsible for the production of G->A transitions in the *K-ras* oncogene that occur in workers with the sentinel neoplasm for vinyl chloride exposure, angiosarcoma of the liver and the biomarkers for these mutations that occur in exposed workers without tumors. These mutant *ras* biomarkers occur in a statistically significant dose-response relationship with regard to cumulative vinyl chloride exposure; however, at any given exposure level, there are seemingly otherwise similar individuals who differ in the occurrence of the biomarkers, suggesting that some genetically determined susceptibility could account for different outcomes despite similar exposures. The presence of the Gln751 polymorphism in XPD appears to explain much of this differential susceptibility. For instance, vinyl chloride exposed workers who were heterozygous for the Gln751 polymorphism were found to have a 1.6-fold increased risk for the occurrence of the mutant *ras* biomarkers and workers who were homozygous for the Gln751 polymorphism were found to have a 2.6-fold increased risk for the occurrence of the mutant *ras* biomarkers, even after controlling for potential confounders including cumulative vinyl chloride exposure, yielding a highly statistically significant allele-dosage effect.[15] These findings are consistent with the fact that the repair of the etheno-guanine adducts produced by vinyl chloride could occur by NER or TCR via XPD-related repair machinery. As noted, amino acid residues in the C-terminal domain of XPD have been implicated in binding to other components of the TFIIH complex, particularly the N-terminal domain of the p44 helicase activator protein.[2] Thus, a change in conformation in the C-terminus of XPD caused by the polymorphism could alter the ability to bind to p44, diminish the activation of its helicase activity and decrease the DNA repair capability of TFIIH for removal of the vinyl chloride induced etheno-guanine adducts resulting in the increase in mutant biomarkers observed at the same levels of exposure in individuals with the polymorphism. These results are further supported by recent studies from other investigators of vinyl chloride workers in China, where the XPD Gln751 polymorphism was found to be statistically significantly associated with increases in non-specific markers of DNA damage as measured by the single cell gel electrophoresis assay.[16]

In summary, the results of the present study provide support for the hypothesis that the position 751 polymorphism in XPD could influence DNA repair capability by altering the structure of the C-terminal domain and disrupting its interaction with other components of the repair machinery. Since this is a relatively common polymorphism in many populations,[3] these conformational alterations and their functional effects could account for a significant amount of the observed variability in DNA repair and hence cancer risk in these populations.

## AUTHOR'S PROFILE

**Paul W. Brandt-Rauf, Dr.P.H., M.D., Sc.D.,** is currently the Dean of the School of Public Health at the University of Illinois at Chicago. In addition, he holds appointments as Professor of Environmental and Occupational Health Sciences in the School of Public Health, Professor of Medicine in the College of Medicine, Professor of Bioengineering and Chemical Engineering in the College of Engineering, Professor of Earth and Environmental Sciences in the College of Liberal Arts and Sciences, and Professor of Public Administration in the College of Urban Planning and Public Affairs. He is also Professor Emeritus of Environmental Health Sciences, Medicine and Earth and Environmental Engineering at Columbia University where he was Chairman of the Department of Environmental Health Sciences until 2008. His major research interest is environmental carcinogenesis, particularly the molecular biology and molecular epidemiology of cancer-related proteins.
